# Supplementary material for: The placental vasculature is affected by changes in gene expression and glycogen-rich cells in a diet-induced obesity mouse model
Source: PLoS One. 2023 Nov 10;18(11):e0294185. doi: 10.1371/journal.pone.0294185 (PMC10637699; doi:10.1371/journal.pone.0294185)
Supplement: S1 Table — (DOCX) [file pone.0294185.s001.docx]

**S1 Table. Gene-specific primer sequences used in the qPCR panel.**

|  | **Primer sequence (5’ → 3’)** | |
| --- | --- | --- |
| **Gene** | **Forward** | **Reverse** |
| *Hif1a* | CACCGATTCGCCATGGA | TTTCTTTTCGACGTTCAGAACTCAT |
| *Hif2a* | GTGACATGATCTTTCTGTCGGAA | CGCAAGGATGAGTGAAGTCAAA |
| *Sod1* | AACCAGTTGTGTTGTCAGGAC | CCACCATGTTTCTTAGAGTGAGG |
| *Sod2* | CCAAGGGAGATGTTACAACTCAG | GGGCTCAGGTTTGTCCAGAA |
| *Gpx1* | AGTCCACCGTGTATGCCTTCT | GAGACGCGACATTCTCAATGA |
| *Hmox1* | CCTTCCCGAACATCGACAGCC | GCAGCTCCTCAAACAGCTCAA |
| *Hmox2* | GGAGGGGGTAGATGAGTCAGA | TCGGTCATGTGCTTCCTTGGT |
| *Pgf* | TGCTGGGAACAACTCAACAGAA | TCTCCATGGGCCGACAGTAG |
| *Cxcl12* | TGCATCAGTGACGGTAAACCA | CACAGTTTGGAGTGTTGAGGAT |
| *Cxcr4* | GAAGTGGGGTCTGGAGACTAT | TTGCCGACTATGCCAGTCAAG |
| *Vegfa* | GCACATAGAGAGAATGAGCTTCC | CTCCGCTCTGAACAAGGCT |
| *Vegfr1* | TTGAAAGAGTCACAGAGGAGGATGAG | TCCGGCAGGTGGGTGATTTCT |
| *Vegfr2* | GTGATCCCAGATGACAGCCA | GGTGAGCGCAGTGTGGTCC |
| *sFlt-1* | GTCACAGATGTGCCGAATGG | TGACTTTGTGTGGTACAATC |
| *Mmp9* | GCAGAGGCATACTTGTACCG | TGATGTTATGATGGTCCCACTTG |
| *Mmp2* | ACCTGAACACTTTCTATGGCTG | CTTCCGCATGGTCTCGATG |
| *Angpt1* | ATCCCGACTTGAAATACAACTGC | CTGGATGATGAATGTCTGACGAG |
| *Fgf1* | CAGCTCAGTGCGGAAAGTG | TGTCTGCGAGCCGTATAAAAG |
| *iNos* | GGAGCCTTTAGACCTCAACAGA | TGAACGAGGAGGGTGGTG |
| *eNos* | TGGAGAGAGCTTTGCAGCAG | GATATCTCGGGCAGCAGCTT |
| *P21* | CGAGAACGGTGGAACTTTGAC | CCAGGGCTCAGGTAGACCTT |
| *Tgfb* | CTTCAATACGTCAGACATTCGGG | GTAACGCCAGGAATTGTTGCTA |
| *Il10* | GCTGGACAACATACTGCTAACC | ATTTCCGATAAGGCTTGGCAA |
| *Tnfa* | ACGGCATGGATCTCAAAGAC | GTGGGTGAGGAGCACGTAGT |
| *Il6* | CCAGAAACCGCTATGAAGTTCCT | CACCAGCATCAGTCCCAAGA |
| *Il1b* | GAAATGCCACCTTTTGACAGTG | TGGATGCTCTCATCAGGACAG |
| *Sp1* | AATCAAACCTTACTCGCCTCTG | GCACATTAGCGACTACTTGAGTT |
| *Ap2* | TTTTTCAGCTATGGACCGTCAC | GAAGTCGGCATTAGGGGTGTG |
| *Nrf2* | TCTTGGAGTAAGTCGAGAAGTGT | GTTGAAACTGAGCGAAAAAGGC |
| *Bach1* | GCCTGAAGAGGTAACGGTTAAA | GCACACTTCGTCAACATTGTC |
| *Actb* | AAGGAGATTACTGCTCTGGCTCCTA | ACTCATCGTACTCCTGCTTGCTGAT |
| *Gapdh* | TGACCTCAACTACATGGYCTACA | CTTCCCATTCTCGGCCTTG |
