# Supplementary material for: The placental vasculature is affected by changes in gene expression and glycogen-rich cells in a diet-induced obesity mouse model
Source: PLoS One. 2023 Nov 10;18(11):e0294185. doi: 10.1371/journal.pone.0294185 (PMC10637699; doi:10.1371/journal.pone.0294185)
Supplement: S2 Table — Placental efficiency (PE) was then calculated as the ratio of fetal:F-placental weights. (DOCX) [file pone.0294185.s002.docx]

**S2 Table. Weights (in mg) of fetuses and F-placentas harvested at E14.5 and E16.5 from mice subjected to the Control, HFD-P, and HFD-PreCP regimens.** Placental efficiency (PE) was then calculated as the ratio of fetal:F-placental weights.

|  | **E14.5** | | | **E16.5** | | |
| --- | --- | --- | --- | --- | --- | --- |
|  | **Control** | **HFD-P** | **HFD-PreCP** | **Control** | **HFD-P** | **HFD-PreCP** |
| **Fetal Weight (mg)** | 316 ± 8 | **347 ± 17*** | 317 ± 18 | 516 ± 56 | 545 ± 37 | 532 ± 48 |
| **n** | 18 | 24 | 24 | 15 | 17 | 17 |
| **F-Placental Weight (mg)** | 39.2 ± 0.8 | 45.0 ± 4.9 | 46.4 ± 2.1 | 50.9 ± 5.4 | 49.9 ± 3.3 | **64.6 ± 4.9**** |
| **n** | 3 | 3 | 3 | 5 | 6 | 4 |
| **PE** | 8.0 | 7.7 | 6.8 | 10.1 | 10.9 | 8.2 |

Data are presented as mean ± SD. *P < 0.0001 vs Controls and HFD-PreCP; **P < 0.005 vs Controls and HFD-P using one-way ANOVA tests with Bonferroni correction.;
