# Supplementary material for: The placental vasculature is affected by changes in gene expression and glycogen-rich cells in a diet-induced obesity mouse model
Source: PLoS One. 2023 Nov 10;18(11):e0294185. doi: 10.1371/journal.pone.0294185 (PMC10637699; doi:10.1371/journal.pone.0294185)
Supplement: Attachment — Submitted filename: Responses_to_Reviewers.docx [file pone.0294185.s003.docx]

**Response to Reviewers**

**Reviewer 1:**

*After thoroughly reading the paper, twice, I found no reason for demanding changes nor corrections of the text. Perhaps it could be summarised a little bit as it is rather long. This notwithstanding I recommend to publish it in its current form*

*We thank the Reviewer for the positive comments!*

**Reviewer 2:**

*I read this manuscript and found it very interesting especially nowadays with the prevalence of overweight and obesity. I have some advice regarding citing many statements both in the introduction and in the discussion. Many statements need appropriate citing.*

*We thank the Reviewer for the positive review. We have now included 8 additional citations (ref #’s 5, 6, 7, 8, 23, 44, 53, and 60) and cited #’s 45, 46 [old #’s 40 and 41] for another statement) in the Introduction and Discussion as recommended.*
